# Supplementary figures and images for: Molecular Characterization of a Rare Case of Bilateral Vitreoretinal T Cell Lymphoma through Vitreous Liquid Biopsy
Source: Int J Mol Sci. 2021 Jun 5;22(11):6099. doi: 10.3390/ijms22116099 (PMC8201094; doi:10.3390/ijms22116099)

Figure S1

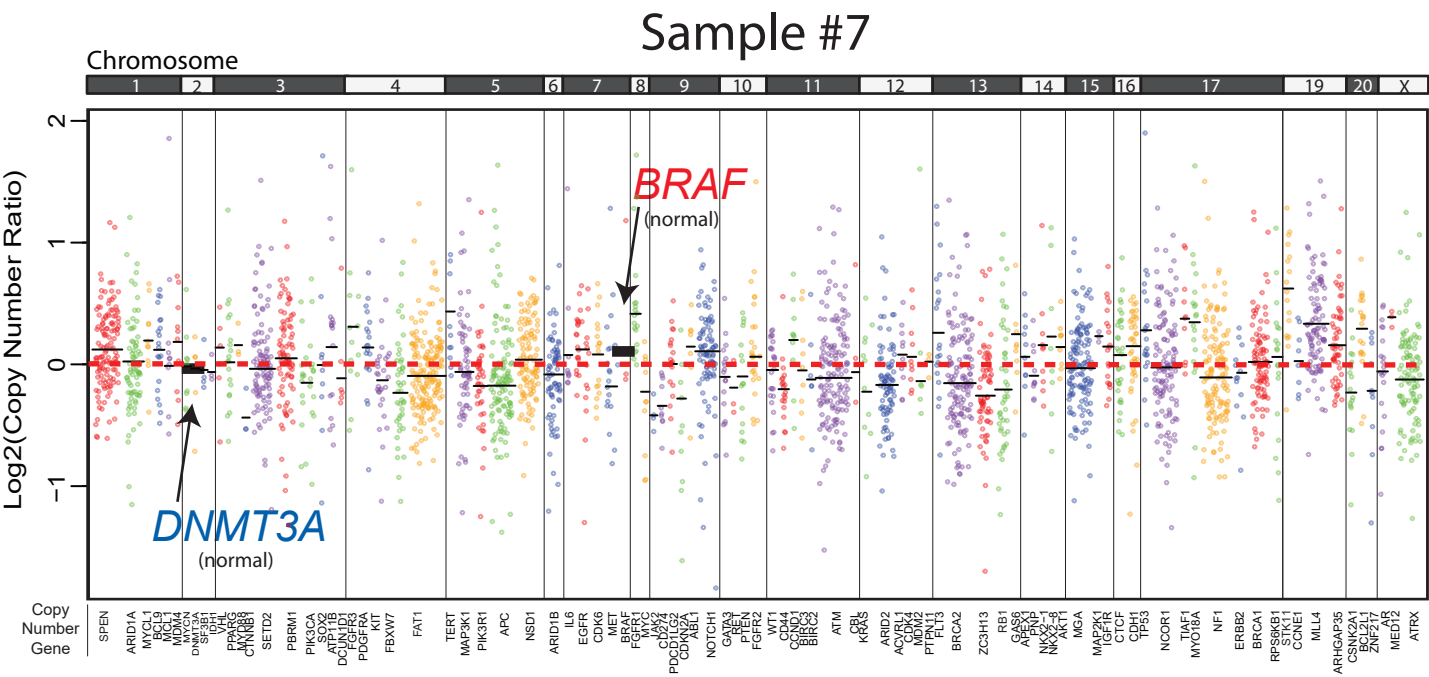

Supplement: Supplementary file 1 [file ijms-22-06099-s001.zip › ijms-1216746-supplementary.pdf]
